# PaperBot: open-source web-based search and metadata organization of scientific literature

**DOI:** 10.1186/s12859-019-2613-z

**Published:** 2019-01-24

**Authors:** Patricia Maraver, Rubén Armañanzas, Todd A. Gillette, Giorgio A. Ascoli

**Affiliations:** 1 Center for Neural Informatics, Structures, & Plasticity; Krasnow Institute for Advanced Study; George Mason University, Fairfax, USA; 2 Bioengineering Department; George Mason University, Fairfax, USA

**Keywords:** Open-source, Microservices, Scientific indexer, Cloud-ready software

## Abstract

**Background:**

The biomedical literature is expanding at ever-increasing rates, and it has become extremely challenging for researchers to keep abreast of new data and discoveries even in their own domains of expertise. We introduce PaperBot, a configurable, modular, open-source crawler to automatically find and efficiently index peer-reviewed publications based on periodic full-text searches across publisher web portals.

**Results:**

PaperBot may operate stand-alone or it can be easily integrated with other software platforms and knowledge bases. Without user interactions, PaperBot retrieves and stores the bibliographic information (full reference, corresponding email contact, and full-text keyword hits) based on pre-set search logic from a wide range of sources including Elsevier, Wiley, Springer, PubMed/PubMedCentral, Nature, and Google Scholar. Although different publishing sites require different search configurations, the common interface of PaperBot unifies the process from the user perspective. Once saved, all information becomes web accessible allowing efficient triage of articles based on their actual relevance and seamless annotation of suitable metadata content. The platform allows the agile reconfiguration of all key details, such as the selection of search portals, keywords, and metadata dimensions. The tool also provides a one-click option for adding articles manually via digital object identifier or PubMed ID. The microservice architecture of PaperBot implements these capabilities as a loosely coupled collection of distinct modules devised to work separately, as a whole, or to be integrated with or replaced by additional software. All metadata is stored in a schema-less NoSQL database designed to scale efficiently in clusters by minimizing the impedance mismatch between relational model and in-memory data structures.

**Conclusions:**

As a testbed, we deployed PaperBot to help identify and manage peer-reviewed articles pertaining to digital reconstructions of neuronal morphology in support of the NeuroMorpho.Org data repository. PaperBot enabled the custom definition of both general and neuroscience-specific metadata dimensions, such as animal species, brain region, neuron type, and digital tracing system. Since deployment, PaperBot helped NeuroMorpho.Org more than quintuple the yearly volume of processed information while maintaining a stable personnel workforce.

## Background

The scientific literature is the principal medium for disseminating original research. The public availability of peer-reviewed articles is essential to progress, allowing access to new findings, evaluation of results, reproduction of experiments, and continuous technological improvement. The biomedical community devotes a substantial investment to extracting knowledge and data from publications to facilitate their reuse towards additional discoveries. Many bioinformatics projects prominently rely on careful data collation, standardization, and annotation, providing considerable added value upon sharing the curated knowledge freely through the World Wide Web.

The ever-rising growth rate of the biomedical literature, however, makes it more and more challenging for researchers to keep abreast of new data and discoveries even in their own domains of expertise. Many labs increasingly rely on publication pointers reported through member email lists or social media. This process carries a substantial risk of missing important pieces of data and is thus unsuitable for comprehensive curation efforts requiring all-inclusive coverage. Moreover, relying on highly clustered networks reduces the opportunity for cross-fertilization among disparate fields of science that could nonetheless share non-trivial elements. A further consequence of this inability to mine the literature effectively is the excessive reliance on fewer, highly cited references and a reduced recognition of the research impact of the majority of worthwhile contributions [1].

Robust tools exist to aid specific tasks related to literature management, especially for reference organization and for content annotation. Systems in the first category create customizable workspaces for choosing, tracking, and formatting citations when writing a manuscript, including commercial solutions such as EndNote [2] and open-source alternatives like Zotero [3], Mendeley [4], and F1000 [5]. Systems in the second category provide the means for highlighting and commenting user-selected portions of an article. Examples include Hypothes.is [6], which allows shared group-wise contributions, WebAnno [7], which suggests annotations learned from user behavior, BRAT [8], which supports complex relations between concepts, and NeuroAnnotation Toolbox [9], which facilitates curation with controlled vocabularies. Additional functionality is offered by SciRide Finder [10], which helps find who is citing a given article.

Nevertheless, none of the above platforms directly solves the open problem of continuously and systematically identifying relevant information for a given domain. This process remains difficult and slow, and it frequently constitutes the most serious bottleneck in populating and maintaining databases and knowledge bases. To alleviate this issue, BIOSMILE [11] was introduced to custom-design PubMed queries; additionally, G-Bean [12] uses the MedLine database for an ontology-graph based query expansion, automatic document indexing, and user search intention discovery. With PubCrawler [13], users can customize PubMed and GenBank queries, and receive later or recurrent updates by email. These tools, however, only search titles, abstracts, and keywords. Often the most fitting expressions to identify the information of interest are technical terms typically found in methods sections or figure legends. Thus, identifying those terms requires full-text searches [14], which indeed have been found to return better and more accurate results than just searching summaries [15].

Other tools have been developed to address this limitation, but none provides a general solution. Biolit [16] downloads all PubMedCentral PDFs using their FTP to increase the knowledge of MedLine database, but is restricted to open-access literature. Omicseq [17] searches specifically for gene names in papers. Available crawlers to acquire web content [18] (but not scientific publications) require prior human annotation to identify new content. Importantly, although most of the above tools were freely released on the web, none open sourced the code, and most are no longer available. After evaluating and reviewing existing models, a set of relevant guidelines were proposed for inclusion in a future framework of digital libraries [19].

The tool we introduce with this work was initially developed to support the growth of and data acquisition for NeuroMorpho.Org, a data and knowledge repository aiming to provide unhindered access to all digital reconstructions of neuronal morphology [20]. In order to identify data of interest, the NeuroMorpho.Org curation team must continuously track the scientific literature to determine if any new articles were published describing neuronal reconstructions. For several years, NeuroMorpho.Org curators mined all publications by manually querying six different publisher portals every month. The queries included appropriate combinations of 80 keywords empirically selected to minimize the number of false negatives (missed articles) and false positives (irrelevant hits). Prior to adopting the system described in this report, all returned titles were matched by hand with PubMed entries in order to retrieve the identifiers and to discard previously found articles. Relevant hits were downloaded, if accessible, and evaluated as relevant or irrelevant based on the presence of target data. Lastly, for each relevant record, the author contact information and essential categories of metadata (e.g. animal species, brain region, cell type, reconstruction software, and publication reference) were extracted before requesting the data. The main requirement motivating this project was the automation of the described manual process, in order to reduce the time of neuronal data acquisition and facilitate the tracking process of the data status (requested, received or released) related to each article. A secondary requirement was the ability to display all new potentially relevant articles since last access through a dynamic web portal. We demonstrate how automating the aforementioned steps substantially increased data yield per unit of time without an expansion of resources expended.

All major publishers have full-text search interfaces that do not require commercial licenses, but each interface is different in terms of programmatic access and output format. As a consequence, full-text searches still require largely manual and error-prone human intervention. To date, most attempts to support these efforts with computer technology have involved ad hoc scripts developed for individual projects. Google Scholar and related search engines can in principle reach all public content on the web, but do not have API, use a non-modifiable, proprietary ranking system, do not return unique identifiers, and often truncate the results (title, authors and dates). Not for-profit search engines only search titles and abstracts (PubMed) or open access publications (PubMedCentral). Overcoming these limitations requires a new system interacting with multiple individual publisher search engines.

Here we introduce PaperBot, a free, extensible, and open-source software program that semi-automates the full-text search and indexing of relevant publications from all major publishers and literature portals for the benefit of any laboratories aiming to identify and extract data from published articles. PaperBot can be installed in servers or personal computers and is designed to be integrated into ongoing knowledge mining projects with minimal effort. This platform differs from and improves on existing literature management solutions by autonomously and periodically finding articles and saving those allowed by the institutional licenses. The results are immediately available for evaluation through a web-based graphical user interface. As curators label each article as relevant or irrelevant, adding or editing metadata, PaperBot stores the entries in real time into an information technology service. Users can annotate entries using free text, ontologies or controlled vocabularies retrieved from a database. The tool could be customized for different projects, each with distinct article relevance criteria and individually specified triaging procedures.

### Application workflow

**Fig. 1:**
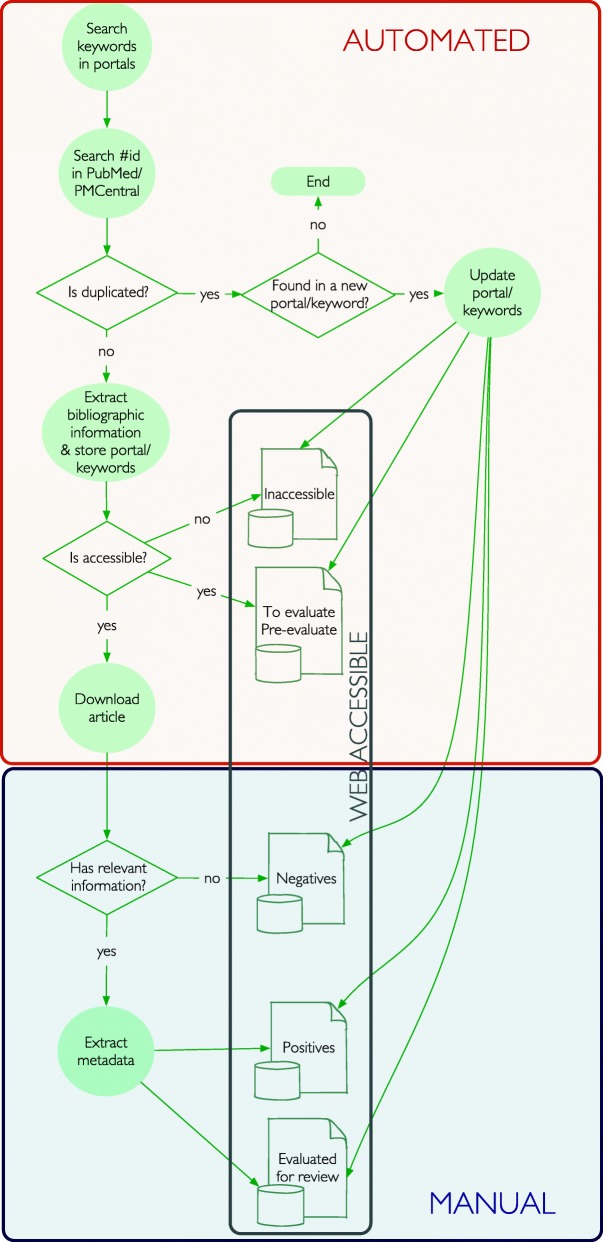
PaperBot article mining pipeline. Flow diagram of the process for identifying peer-reviewed publications that contain data of interest

PaperBot performs automated, periodic, full-text searches for scientific publications and provides an ergonomically effective web interface for their annotation (Fig. 1). The configurable crawler selects and gleans content from multiple journal portals based on user-defined Boolean combinations of search terms, and extracts bibliographic details including title, journal reference, author names, and email contact. Data retrieval makes use of application programming interfaces (API) where available (Elsevier/ScienceDirect, Springer, Nature, and PubMed/PubMed Central). Publishers not providing API access (Google Scholar and Wiley) could be scraped within the limits allowed by the terms of use. The scraper portion of the software is turned off by default, but users can choose to activate it after verifying each publisher’s policy.

Recurrent searches often return duplicate articles from different portals (e.g. Elsevier and PubMed Central) or from the same portal at different times. To identify duplicates, PaperBot compares each article found against all previous results using three parallel methods: exact match of PubMed or PubMed Central identifier (PMID or PMCID), exact match of digital object identifier (DOI), and approximate match of titles using the Jaro-Winkler distance [21] with a precision of 0.85. We empirically found this threshold to ensure robustness with respect to non-uniform representation of special characters across portals and variable trimming of article titles above certain character lengths. PaperBot merges and updates all information retrieved from duplicate articles, including detected keywords and search portals, as well as newly added data (e.g. when articles receive a PMID several months after publication).

Next, PaperBot autonomously attempts to download the article PDF using the pointer provided by the CrossRef registration agency. The PDF accessibility depends on the article open access status or on the institutional/personal subscription to a given journal, typically based on the server’s IP address where PaperBot is running. Accessible articles are stored in an *Evaluate* collection, whereas articles whose PDF cannot be downloaded are saved in an *Inaccessible* record collection. The software will automatically recheck these inaccessible records in all future searches as some articles may become accessible later from the same or different sources due to delayed open access release or new journal subscriptions. When the PDF of a past inaccessible article is eventually acquired, PaperBot moves the corresponding record to the *Evaluate* pool.

All articles in the *Evaluate* collection can be reviewed and annotated. While this step requires human interaction, the ergonomically-optimized web interface of PaperBot makes the curation process easy, fast, and robust. Users can deem each article relevant (*Positive* collection) or irrelevant (*Negative* collection) following project-specific criteria. Alternatively, unjudged articles can be moved to a stand-by *Review* collection accompanied by personal notes to allow further examination. This option allows multiple users to examine a single article and/or to open an on-line discussion about its relevance. Lastly, PaperBot prompts and facilitates user annotation according to a customizable collection of metadata dimensions specified based on project needs or investigator preferences.

While in this work we illustrate the successful application of PaperBot to NeuroMorpho.Org, most database and knowledge base project also rely on literature content curation and can thus similarly benefit from this tool. Just among useful neuroscience resources [22], for instance, ModelDB [23] contains simulation-ready computational models spanning a broad range of biophysical mechanisms at the neuronal and circuit level. The BioModels repository [24] offers a complementary focus on biochemical kinetics and molecular cascades. NeuroElectro [25] provides literature-extracted values of common electrophysiological parameters from major neuron types. CoCoMac [26] is a connectivity database of the macaque monkey cortex. Other similar projects include BrainInfo [27], SenseLab [28], Wormatlas [29], The Brain Operation Database [30], Open Source Brain [31], and Hippocampome.Org [32]. At the human whole-brain non-invasive imaging, NITRC [33] serves both as a neuroimaging data repository and a portal to find neuroinformatics tools and resources. A prominent project in this subfield is the Alzheimer’s Disease Neuroimaging Initiative (ADNI), which provides extensive longitudinal data on this pathology’s biomarkers, including molecular diagnostics, brain scans, and cognitive testing [34]. Other examples include the Autism Brain Imaging Data Exchange (ABIDE) [35], which shares functional and structural magnetic resonance imaging datasets with corresponding phenotypic information, and the Open Access Series of Imaging Studies (OASIS) [36], offering data sets of demented and non-demented subjects.

## Implementation

We designed PaperBot to be flexible, extensible, and ready to use. This was achieved in part by implementing a microservices architecture [37]. The key idea is to structure the application as a collection of loosely coupled services that implement business capabilities. Each service can be deployed in isolation independent of the others, allowing the end user to configure the platform according to their needs. The services communicate in a light-weight manner using a representational state transfer (REST) web API [38]. REST architectural style transfers a representation of the data between components. All of the services are written in Java, although the design allows the integration of different languages for additional services. We used a NoSQL database, MongoDB, due to its scalability in clusters. MongoDB solves the mismatch between the relational model and the in-memory data structures, is schema-less, and naturally works with data aggregates [39].

Specifically, the system is composed of three databases that store all the information, a web interface, and five core web services, each of which runs in an embedded servlet container (Fig. 2). We describe each component below.

1. *Portal & Keywords database* stores the configuration used to access the publishers’ portals, such as URLs, access tokens, configured search terms, start date for the query, and a flag to (de)activate each portal for the next search period/run. This database also records an activity log detailing the start/end times and output status for each execution, namely whether the search returned an error, was interrupted by the user, or finished successfully.

**Fig. 2:**
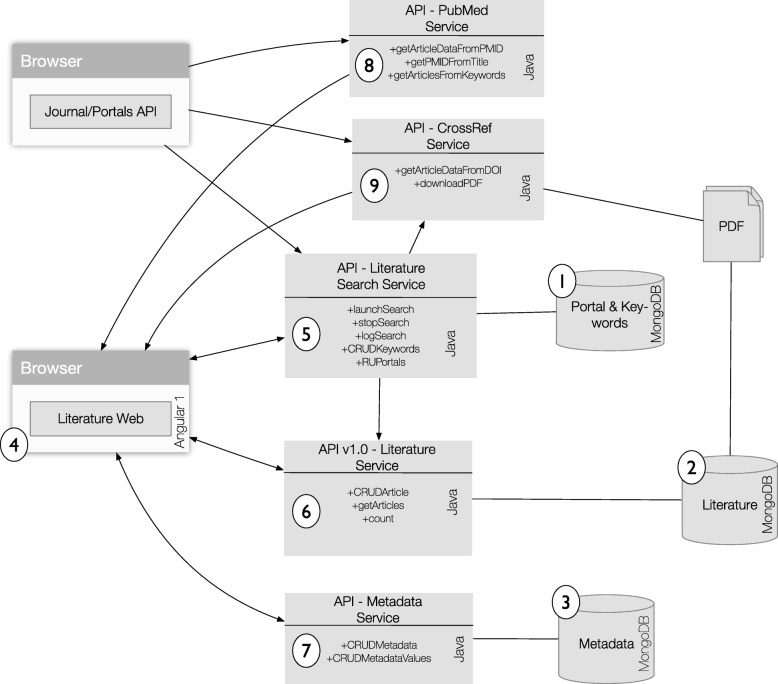
PaperBot microservices information flow. Rectangles represent web applications running an embedded servlet container and its main methods (CRUD is the acronym for Create, Read, Update, and Delete). The arrows represent the flow direction of the data between services. Circled numbers points to the discussion on each component’s specifics included in “Implementation” section of the text
2. The *Literature database* stores bibliographic information of every publication recorded in PaperBot: PMID, DOI, title, journal reference, publication date, author names, and corresponding email when available. Each record also includes additional information about the associated search parameters, such as the specific portal and keywords that identified the article, the date it was found, and the date the publication was evaluated by a user.
3. The last database, *Metadata database*, gathers all extracted metadata annotations from each publication. It is designed to support as many metadata categories as a user requires, and with a variety of types, such as integers, lists, sets, strings, and nested lists. Values for each metadata categories can be expressed as free text or delimited by the pre-configuration of a controlled vocabulary or ontology. Two metadata categories are included by default: a Boolean field, named ‘IsMetadataFinished’, to record whether the user has completed the annotation of all metadata from a publication; and a free-text field, ‘Note’ enabling users to add personalized information or comments to each record.

**Fig. 3:**
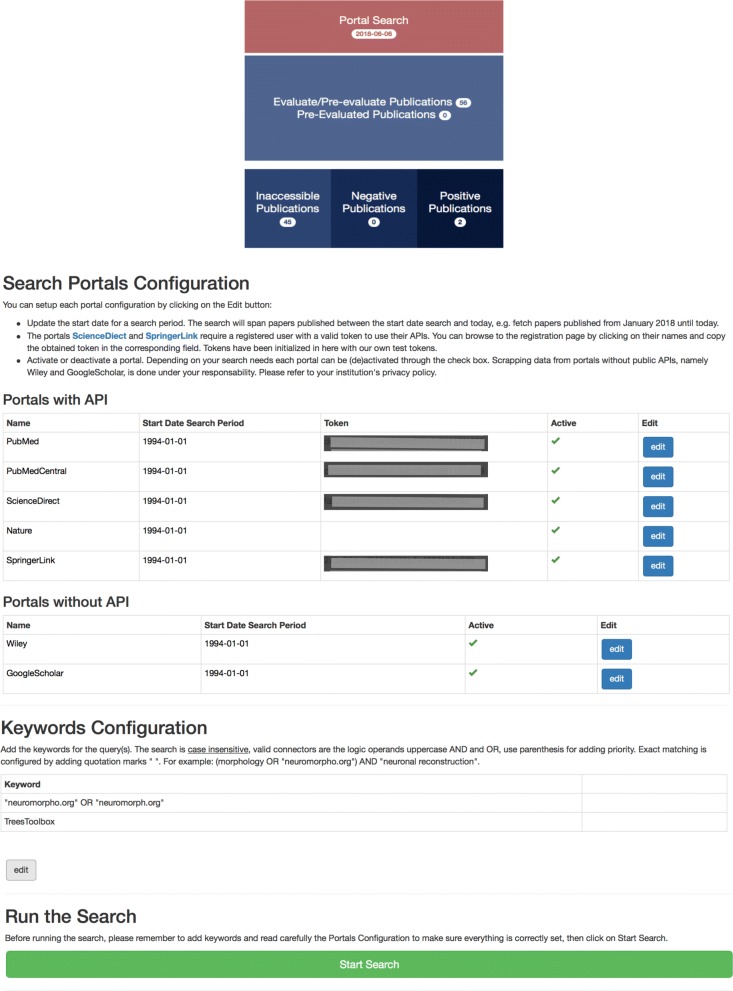
PaperBot portal interface. Top: Principal menu. Bottom: Search configuration interface

**Fig. 4:**
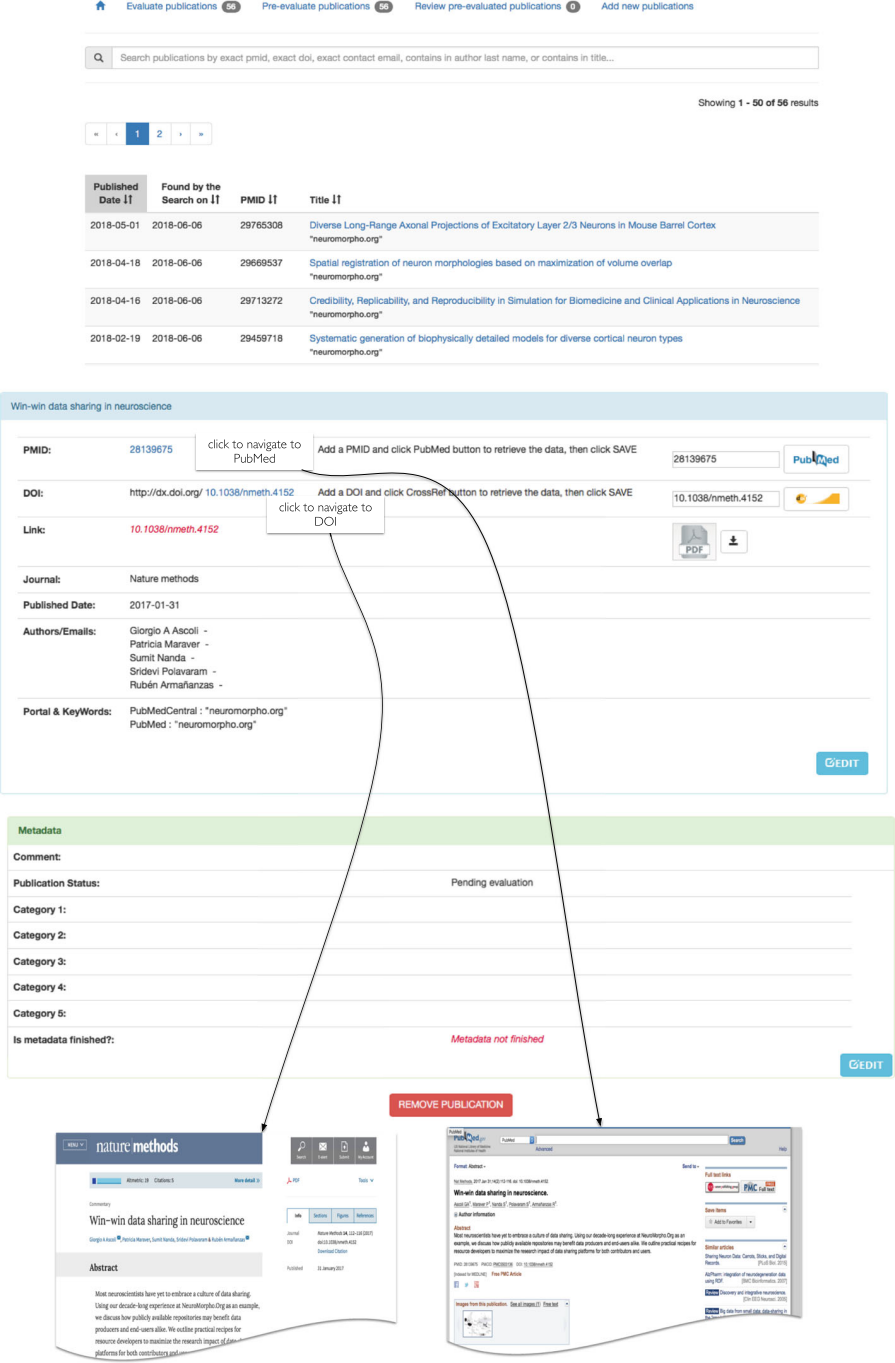
PaperBot portal interface. Top: List of article publications found by the search. Bottom: Article interface containing the bibliographic data and metadata curation interface to annotate the publication
  A common interface allows users to browse the evaluated publications as well as those to be evaluated based on their titles and keywords (Fig. 4 Top). Extended lists are paginated and can be sorted by publication date, identifier or title, as well as filtered by publication identifiers, titles or authors. Clicking on any title opens a new page with the detailed bibliographic information, metadata annotations if any, and the option to update and/or remove that publication (Fig. 4 Bottom). The metadata selection is highly modular, allowing customization of the annotation settings and terms by updating the HTML source code. In addition to the articles identified by the automatic search, the interface also enables the user to add a new publication semi-manually using a PMID or DOI or manually by inserting all relevant fields

The PaperBot web interface provides user-friendly access to the system’s features and publication inventory. The interface to browse, inspect, and annotate a publication is depicted in Figs. 3 and 4. The following are the key components of the web interface.

4) The *Literature Web* allows the user to orchestrate all interactions among the rest of services. It is written in Javascript/CSS/HTML and designed using AngularJS, a development framework created by Google for building mobile and desktop web applications. The front page includes links to the search configuration, the evaluation and assessment of publications, and a direct access to the three major collections: positive, negative, and inaccessible articles (Fig. 3 Top). The search configuration facilitates the management of the search portals, search time span, and keywords. Functions include launching and stopping searches, displaying information on undergoing searches or previous activity logs, and an option to reset the whole database in case of need (Fig. 3 Bottom).

The remaining five services constitute the inner engine of the system. Extended information on each service’s functionalities follows.

5) The *Literature Search Service* is responsible for searching, retrieving, and storing bibliographic information from different publisher portals, and as such can be considered PaperBot’s core. Since different publishers use different search interfaces with distinct input requirements, this service maps instructions from the user to the required formats of the individual portals. Specifically, the Literature Search Service reads the portal-specific configurations for search expressions, search dates, and search portals from the Portal & Keyword database. Those query parameters are fixed within a search but users can change them from one search to another. The service searches for (case insensitive) terms or exact phrases (by adding quotation marks “"), combined via logical AND/OR (uppercase) operands, where parentheses are used to add priority. An example of the search is: (morphology OR “neuromorpho.org") AND “neuronal reconstruction". Certain aspects of the capability depend on the portal being queried: for instance, the operand NOT can be currently used by all portals except for Google Scholar, which does not support it. The APIs use the lemma of the word for searching: as a case in point, translating the word ‘neurons’ into ‘neuron OR neurons’. PubMed performs MeSH searches, automatically expanding a word into the set of all its matching synonyms within the controlled vocabulary. In all cases, the returned entries are displayed in the PaperBot web page as a table that can be sorted by PMID, title, published date, or search date.
  The *Literature Search Service* communicates with the *Literature Service* to store the bibliographic information gleaned from the publisher portals. For each publication acquired, the Literature Search Service records the information of each query (which portal and keywords identified the publication) and then calls the *PubMed Service* to retrieve complementary data, namely its associated identifier (PMID or PMCID) and the corresponding author’s email. With these data, the *Literature Service* either saves the publication as a new entry or updates an existing entry. If the PDF file of a new publication was not locally saved, the service will pass its data to the *CrossRef Service* asking for the download. The download status of the file is updated based on the success of the download process. When a publication is pay-walled by a publisher, the download status will depend on the credentials associated to the PaperBot host machine. If the file ended up being not reachable, the article is moved to the ‘Inaccessible’ collection. In future searches the service will check again if the download is possible. A factory pattern is used to implement the functionality of the different portals. We unify the searches for the user, since every API works differently: to build the queries we concatenate each portal required parameters with the root URL and translate the keywords to the portal requirements. For example, Nature queries use the character ‘+’ between words instead of white spaces; certain APIs return XML while others return JSON; some publisher portals permit filtering by year and month, others only by year (PaperBot allow complete date filtering by year, month, and day, comparing the published date of the article with the filtered date provided by the user and discarding the articles out of range); scraping is needed when no API is provided, using a headless webpage library that waits until the page fully loads, then reads the html tags and its content. When no results are returned from the query, the process continues to the next query.
  Every keyword query is associated with a specific user-defined collection where the data are saved in the database. This design element provides additional flexibility: while PaperBot was created to aid dense literature coverage in a given domain, the collection used to save the articles depending on the query functionality could be exploited by other projects with different needs, for instance where one or two references may be sufficient to support a relevant piece of knowledge. In that alternative scenario, once the references are identified, the collection associated with the query could be updated to an “already identified" status.
6) The *Literature Service* manages all bibliographic data stored in the Literature database. It is the only process that has access to this information. Any other service in the system must retrieve these details by using the common API. This design provides isolation between databases and the rest of the system. The Literature Service has methods to create, update, delete, read, and count publication states, manage list of items, and store or update the result of different searches. This service receives every article found by the search, sifts through all the collections in the database comparing PMID, PMCID, DOI, and approximate title (matched using the Jaro-Winkler distance). If the article is not found, the new article is saved; otherwise the service checks if any of the information elements retrieved by the search (DOI, PMID, published date) is missing from the stored article in the database and fills it as needed; this is especially important when the same manuscript is first detected as a BioRxiv preprint and later as peer-reviewed article. The service also examines the keywords and portal name corresponding to each article in the form of an array of objects, using an update operation similar to addToSet: namely, adding a value to the array unless the value is already present, thus avoiding the creation of duplicates.
7) The *Metadata Service* is in charge of the annotation of data extracted from the publications. Metadata values could come from natural language concepts or retrieved from a controlled source such as a database or ontology. The service is designed to manage any kind of object allowing full customization of its configuration. To provide this flexibility, the code is implemented using weak typed data (type Object in Java). Although this is more versatile, it relies on the web interface to maintain database coherence.
8) The *PubMed Service* accesses NCBI’s PubMed and PubMedCentral programming interfaces to retrieve PMID and PMCID identifiers and extended bibliographic information using the title of the document. The service returns complete publication records to the rest of services, including manual queries to add new publications based on a PMID or PMCID through the Literature web interface. When using a title to obtain identifiers, NCBI typically returns multiple articles due to the MeSH expansion of every term; the PaperBot PubMed Service extracts each of the returned articles information with distinct API calls and identify the best match based on Jaro-Winkler distance with a hard threshold of 0.9.
9) *CrossRef Service* queries the CrossRef registration agency of the International DOI Foundation to fetch the unique resource locator (URL) of a publication’s PDF by means of its DOI. The service receives a request from the *Literature search service* for the associated URL of a newly found publication. The service also interacts with the *Literature web* when a user manually adds a new publication using its unique DOI, or requests update information that may be missing by clicking the CrossRef button. Currently the article PDF files are saved in the database as blobs and loaded from the web page for user access. This choice implies higher RAM demands for larger numbers of articles. If the project does not require PDF annotation and a high load is expected, we recommend storing the article files in the hard drive instead.

## Results

As a representative testbed of PaperBot, for over two years we harnessed the described functionalities in support of the data sharing repository NeuroMorpho.Org. The scope of this popular neuroscience resource pertains to digital reconstructions of brain cells morphology described in peer-reviewed articles [20]. Specifically, the mission of NeuroMorpho.Org is to provide free community access to all such data that authors are willing to make publicly available. Achieving such dense coverage of the existing data requires assiduous screening of the relevant scientific literature. In fact, the project’s success hinges on the systematic search for, and effective identification of any new publications containing digitally reconstructed neuromorphological data, followed by a collegial invitation to the corresponding author(s) to share their dataset. This process entails a complex battery of combined keyword queries over several full-text search engines followed by the critical evaluation and annotation of every article found. Managed manually until recently, these operations constituted a labor-intensive, time-consuming, and error-prone bottleneck for NeuroMorpho.Org. A similar situation still applies to many other biomedical knowledge base and database curation projects.

**Fig. 5:**
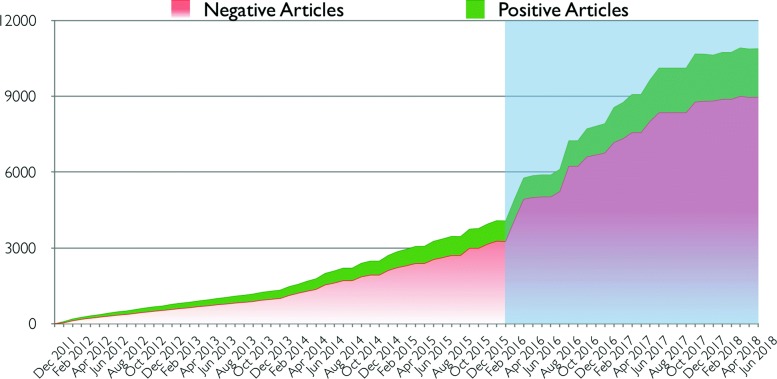
Trends in article search and evaluation for the neuroscience project NeuroMorpho.Org. Cumulative curve of articles mined and assessed as relevant (green) and non-relevant (red) in monthly increments (with bi-monthly labels). The blue shadow highlights PaperBot usage over a 2.5-year period as compared to the previous manual pipeline over a 4-year period

In January 2016 we replaced the above-described manual procedures with a customized instance of the PaperBot tool. In the last four years of manual handling (January 2012 to December 2015) we processed 3238 articles (∼800 per year). In contrast, during the two years following the switch, we found 8207 articles (∼4100 per year). Thus, deployment of PaperBot increased throughput over five-fold (Fig. 5) while maintaining a constant workforce in the evaluation team.

Interestingly, the articles PaperBot identified included 3905 pre-2016 publications not previously detected by the manual system largely due to the necessarily more limited keyword selection suitable for manual searches. Specifically, automation enabled the increase of the number of queries per portal from 80 to more than 900 keyword combinations, which would be practically impossible for a human operator. A team of curators is still involved with carefully evaluating each article and, for every positive hit, extracting metadata such as animal species, brain region, and cell type. However, PaperBot now freed these personnel from taxing and uninteresting tasks such as periodic monthly searches, duplicate detection, and bibliographic information extraction. This resulted in channeling more meaningful effort into the increased volume of article evaluation. The full NeuroMorpho.Org bibliography is openly available at http://neuromorpho.org/LS.jsp where it can be explored and analyzed by year, by data availability status, and by experimental metadata (animal species, brain region, and cell type). Moreover, NeuroMorpho.Org literature data can be programmatically accessed via API at http://neuromorpho.org/api.jsp.

Additionally, by allowing much more complex search queries, the adoption of PaperBot reduced not only the number of false negatives (missed articles), but also that of false positives (irrelevant articles), further increasing the yield of the annotators’ efficiency. The user friendly web interface greatly facilitated both article evaluation and metadata annotation. In particular, the ability for all team members to access the same information aids the collegial resolution of "close calls" while providing a unified management system for querying articles, requesting data, and annotating metadata in parallel. At the same time, extensive usage by multiple team members also ensured robust testing and multi-perspective ergonomic optimization.

Remarkably, we found that exclusively relying on one or two portals was insufficient to track all the articles: in other words, the strategy of running the queries in parallel on all available publisher portals and search engines was instrumental to achieve dense literature coverage (Table 1). From a total of 2637 articles confirmed to be relevant for NeuroMorpho.Org, 1959 (∼75%) were found only by one portal, indicating that the multiple sources were largely complementary. This is not unexpected with respect to the non-overlapping databases of competing publishers (e.g. SpringerLink and Elsevier ScienceDirect), but it may come as a surprise when considering umbrella search engines. The explanation for these findings reflects the limitations described in the “Background” section: PubMed does not search the full text, PubMedCentral only taps into open access publications, and Google Scholar is typically delayed in indexing new articles especially relative to the publisher’s “ePub ahead of print" publication date. Furthermore, the coverage afforded by the broad selection of portals interacting with PaperBot proved to be comprehensive, since the number of inaccessible articles remained systematically below 1% (26 vs. 2637 confirmed relevant articles as of November 2018).

**Table 1:** Portal searches results

Portal	Articles found by multiple portals	Articles found only by a single portal
PubMed	82	29
PubMedCentral	540	1320
Google scholar	372	315
Nature	52	4
Wiley	226	186
ScienceDirect	82	87
SpringerLink	81	18

Uniqueness and redundancy in over multiple portal searches

The integration of PaperBot into the NeuroMorpho.Org pipeline also opened the possibility of new kinds of searches to monitor the impact of the project in the scientific community. Leveraging the customizable design of PaperBot, we launched an automatic periodic query for *“NeuroMorpho.Org”* as the keyword and assessed the records retrieved for type of data usage. The search identified 389 studies that directly utilized neuronal reconstructions downloaded from the repository, plus an additional 216 articles that simply cited or described the resource. The results of these searches and the corresponding identified references are publicly accessible through http://neuromorpho.org/LS_usage.jsp.

In summary, PaperBot vastly improved the search for relevant data from peer-reviewed publications, more than quintupling the yearly number of identified articles for NeuroMorpho.Org while eliminating human involvement in tedious, no-value-added steps. This tool, in whole or via appropriate combinations of its modules, could help other laboratories and projects improve their data acquisition pipelines and information curation workflows.

## Conclusions

Science, once the vocation of a few, is now standing on the shoulders of many, with estimates of over 100 million scholarly documents available on the web [40]. This deluge of knowledge is practically impossible for individual researchers or single labs to scan comprehensively or to review except in the tiniest of proportions. In its entirety, such massive literature is out of reach even for the largest organizations if taken individually: the National Library of Medicine and PubMed "only" index 28 million records, Semantic Scholar reaches 39 million, and Scopus also falls short at 70 million. Major publishers such as Elsevier, Springer, and Wiley provide search tools to query "their" journals, leaving the non-trivial task to collate and organize the results to the customers. The two largest literature crawlers today, Google Scholar and Microsoft Academic Search, have in many cases no access to full text content, in addition to using proprietary algorithms to rank and filter the resulting output.

Many modern data-driven projects can benefit from automating the identification and digestion of relevant material from this gargantuan glut of information. The software tool introduced here fills that automation need by providing an open-source, expandable, and customizable server/client platform to monitor and manage past, present, and future scientific publications. PaperBot is not linked to any particular central server, runs stand-alone, and can be installed locally or in the cloud. PaperBot is designed to be permanent and self-updating: all content identified always remains stored for user access at any time; meanwhile, autonomous background searches periodically scan the literature to update the content with the most recent publications.

The rapid growth of the annotated literature database enabled by PaperBot, as demonstrated by the application to NeuroMorpho.Org, opens exciting new prospects. The large number of already-archived publications identified as positive or negative, along with their keyword combinations and bibliographic information (journal source, author identity, etc.), can be analyzed and mined to improve further the search process and future keyword selection. Moreover, the accumulated data may be exploited to train state-of-the-art machine learning algorithms for ranking newly found items by their potential relevance to the project. Further developments may entail machine annotation of metadata dimensions through the automatic extraction and classification of textual entities.

Besides its direct utility for the maintenance and growth of literature-dependent databases, PaperBot may prove useful for additional applications. Students could use it to monitor subfields of interest while seeking suitable topics for their projects. Researchers will have a mean to detect cliques of peers working on similar techniques. Principal investigators might strengthen their grant proposals by demonstrably identifying critical knowledge gaps in the community. Conversely, funding institutions could make use of thorough and continuous literature scans to assess the impact of ongoing and past projects at evaluation periods.

## Availability and requirements

PaperBot and its code are licensed under the three clause BSD license [41]. The source-code is deposited on GitHub available at https://github.com/NeuroMorphoOrg and a functional demo of PaperBot’s implementation is accessible for public testing at http://PaperBot.io.

**Project name:** PaperBot

**Project home page:** http://PaperBot.io

**Operating system:** Platform independent

**Programming language:** Java, Javascript, HTML, CSS

**Other requirements:** MongoDB

**License:** Three clause BSD

**Source code:** https://github.com/NeuroMorphoOrg
